# Biomechanical stability of hernia-damaged abdominal walls

**DOI:** 10.1038/s41598-023-31674-w

**Published:** 2023-03-27

**Authors:** Ali Karrech, Hairul Ahmad, Jeffrey M Hamdorf

**Affiliations:** 1 School of Engineering, University of Western Australia, Perth, Australia; 2 UWA Medical School, University of Western Australia, Perth, Australia; 3 Perth Hernia Institute, Murdoch, Australia

**Keywords:** Biomedical engineering, Experimental models of disease

## Abstract

Hernia occurs when the peritoneum and/or internal organs penetrate through a defect in the abdominal wall. Implanting mesh fabrics is a common way to reinforce the repair of hernia-damaged tissues, despite the risks of infection and failure associated with them. However, there is neither consensus on the optimum mesh placement within the abdominal muscles complex nor on the minimum size of hernia defect that requires surgical correction. Here we show that the optimum position of the mesh depends on the hernia location; placing the mesh on the transversus abdominis muscles reduces the equivalent stresses in the damaged zone and represents the optimum reinforcement solution for incisional hernia. However, retrorectus reinforcement of the linea alba is more effective than preperitoneal, anterectus, and onlay implantations in the case of paraumbilical hernia. Using the principles of fracture mechanics, we found that the critical size of a hernia damage zone becomes severe at 4.1 cm in the rectus abdominis and at larger sizes (5.2–8.2 cm) in other anterior abdominal muscles. Furthermore, we found that the hernia defect size must reach 7.8 mm in the rectus abdominis before it influences the failure stress. In other anterior abdominal muscles, hernia starts to influence the failure stress at sizes ranging from 1.5 to 3.4 mm. Our results provide objective criteria to decide when a hernia damage zone becomes severe and requires repair. They demonstrate where mesh should be implanted for a mechanically stable reinforcement, depending on the type of hernia. We anticipate our contribution to be a starting point for sophisticated models of damage and fracture biomechanics. For example, the apparent fracture toughness is an important physical property that should be determined for patients living with different obesity levels. Furthermore, relevant mechanical properties of abdominal muscles at various ages and health conditions would be significant to generate patient specific results.

## Introduction

Hernia is a common pathology that is believed to arise from biomechanical and biochemical alterations within a specific muscle or a group of muscles. More than 20 million surgeries are performed annually around the globe to correct hernias^1^. While these surgeries might be considered routine procedures, the cumulated recurrence risk can range from 15 to 35% after hernia repair^2^, according to a large scale study of ventral hernias that covered 2.5 million people over a period of 4 years. Predicting the size of hernia defect that is significant enough to require surgical correction, as well as predicting the hernia defect size requiring mesh reinforcement and determining the optimum positioning of mesh implants within the abdominal wall (AW) for adequate mechanical reinforcement should reduce the risk of hernia recurrence. Experiment-informed three-dimensional (3D) computational modelization can be instrumental to objectively predict the anatomic functioning of abdominal muscles. For example, the finite element method (FEM) has been used to simulate the genesis of inguinal hernia and demonstrate Keith’s long-standing conjectures^3^ regarding the diminishing risk of hernia with the increase of muscular mass^4^. In addition, FEM has been used to examine the constitutive behaviour of various tissues in the AW^5^. More recently, holistic models based on FEM have been developed to simulate the biomechanics of AWs subjected to local weaknesses in various locations seeking to address challenging scenarios of hernia development^6–9^. For example, Tuset et al.^9^ concluded that intestinal stoma location has no significant impact on the resulting stress and strain distributions except when it is located in the linea alba. As a predictive modelling tool, FEM can complement clinical research and facilitate the surgeons’ decisions. For example, it can be instrumental to visualise, optimise and test hernia repair scenarios before operation given the large number of patient-personalised solutions that are conceivable. A careful survey of the literature reveals that there are no numerical studies that investigate the physics of hernia repair in a systematic manner. Therefore, this paper showcases the potential of FEM in addressing the reinforcement of hernia-damaged AW.

In the present study, we developed a finite element computational setup to predict the response of a typical abdominal wall (AW) to intra-abdominal pressure (IAP) and investigate the effects of AW integrity on the overall biomechanial behaviour. AW integrity has been considered as an experimental factor by examining abdominal muscles with and without hernia-related damage. Where damage has occurred, the influence of repair mode on the response has been investigated by varying the position of the mesh through the AW. IAP is known to depend on the health conditions of the patient (e.g. obesity, pregnancy). For example, the healthy weight individuals have IAP varying from 0.66 to 0.93kPa while those with clinically severe obesity have IAP measurements ranging from 1.2 to 1.9kPa^10,11^.

There are four essential sections in this paper. The first section is introductory and includes a brief literature survey on AW modelling using FEM. The second section describes the numerical model and explains the constitutive relations used for integration. The third section is dedicated to the numerical results and their analysis with special focus on the significance of damage induced stress concentration in abdominal muscles. The fourth section draws the conclusions of this study and summarises the main interpretations.

## Methods

### Description of the model

**Figure 1:**
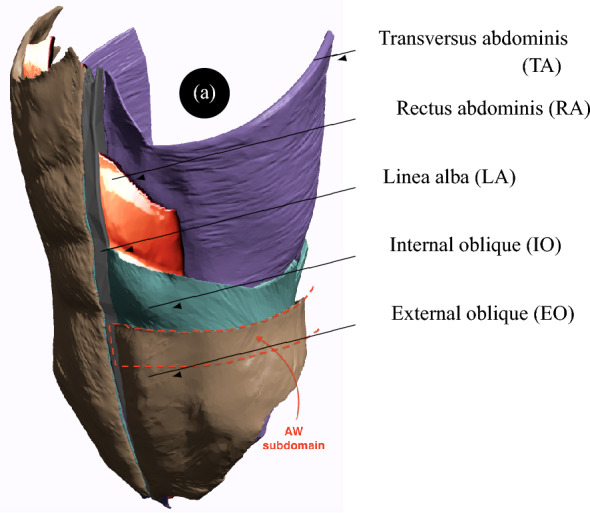
Anatomic geometry of AW where the upper regions of the left transversus abdominis, rectus abdominis, internal oblique, and external oblique were removed to better illustrate the assembly.

**Figure 2:**
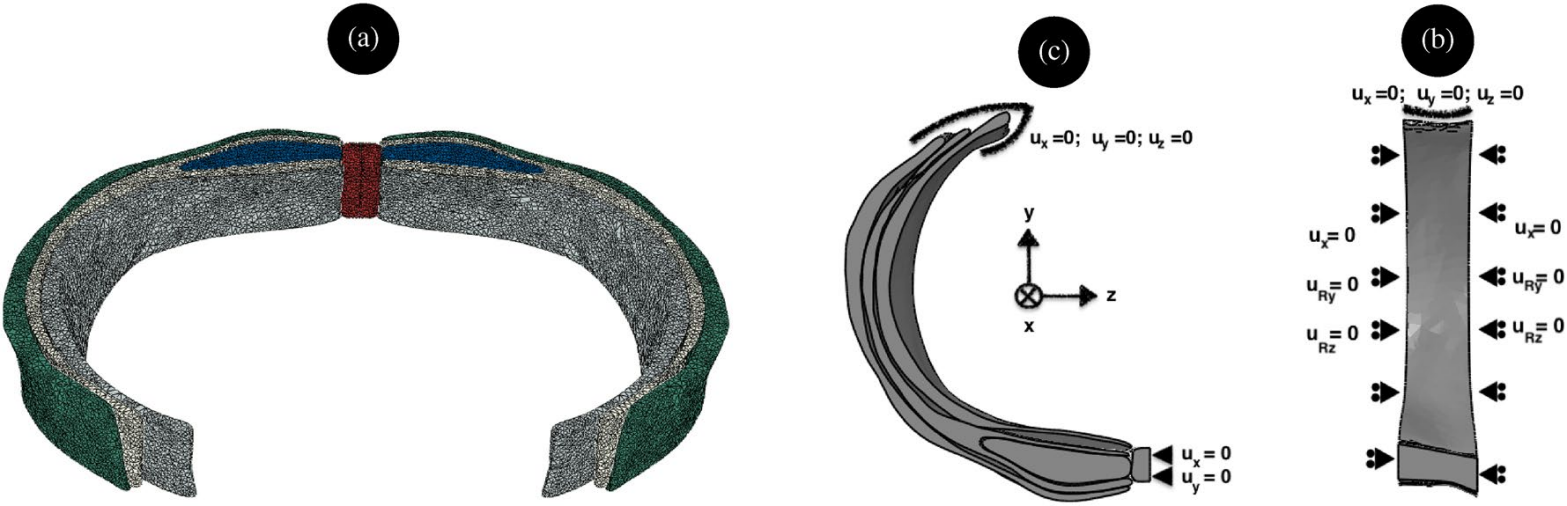
(**a**) Representation of the finite element mesh (**b**) top view of the AW subdomain with boundary conditions, and (**c**) side view of the AW subdomain with boundary conditions.

The geometry of the model is depicted in Fig. 1 representing the reconstructed abdominal wall. This geometry was obtained by superimposing the external oblique (EO), internal oblique (IO), rectus abdominis (RA), transversus abdominis (TA), and linea alba (LA) muscles and aponeuroses (for pragmatic purposes, we combined muscles and aponeuroses functionally), where LA receives the oblique and abdominis muscles and runs down the midline of the abdomen. The geometries of these muscles were based on micro-computed tomography scans available in the BodyParts3D anatomy database^12^. The 3D models of the muscles were then refined using Meshmixer^13^ to create smooth triangulated surface geometries and store them in the object file format. Without merging them in Meshmixer, the obtained surface triangulations were exported to the general purpose finite element software Abaqus^14^ as individual meshes in the standard tessellation format (STL). In abaqus, the individual STL tessellations were imported as orphan surface meshes. A 3D geometry with added volume was then created from each orphan mesh – this geometry can be manipulated as a standard computer-aided engineering part in Abaqus. Extruded cuts were applied to the geometrical parts representing the abdominal muscles to isolate an assembled subdomain of interest for hernia calculation in the umbilical region. Tetrahedral elements were used in the isolated subdomain for subsequent analyses. In the subdomain isolating the umbilical region, 32125, 32205, 14111, 30099, and 2844 elements were used for the EO, IO, RA, TA, and LA muscles, respectively (Fig. 2a).

At the midline of the subdomain of interest, LA was prevented from translating vertically (x-axis) and circumferentially (z-axis) and left free to translate radially to represent the symmetry conditions. The ends of the EO, IO, and TA muscles in the lumbar zone were prevented from moving by applying *encastré* boundary conditions (Fig. 2b). The bottom and top surfaces of the subdomain were left free to translate radially and circumferentially and to rotate around the vertical axis; all other degrees of motion were prevented (Fig. 2c). These conditions suggest that the superior and inferior surfaces act like planes of symmetry. Initially, the tissues were assumed to be free of active work and pre-stresses. The internal surface of the AW was subjected to an internal pressure of 0.8kPa, representing the IAP of an average normal body group person.

*Surface-to-surface contact* models were used to predict the mechanical interactions between the muscles. There are three interfaces of mechanical interaction, which are between the TA and IO, the IO and RA, as well as the IO and EO. These interfaces were modelled using a *finite sliding* formulation without *geometrical adjustment* or *surface smoothing*. The *tangential contact* within these interfaces was considered *frictionless*. The linea alba was connected to the above muscles using *tie* constraints, where LA plays the role of the *master surface*. This constraint ensures that the translational and rotational degrees of freedom are equal on the tied interface.

Where a hernia occurs in the AW, it was modelled as a damage zone of diameter 8 mm throughout the wall. The mesh implant used to simulate hernia repair or wall reinforcement was considered to have a Young modulus of 8 MPa, based on the experimental data of Kirilova et al.^15^. Poisson’s ratio of the mesh implant was 0.3 according to the experimental data of Szepietowska and Lubowiecka^16^. Mesh implants usually come in small size 4.3 cm or medium size 6.4 cm^17^; given the symmetry boundary conditions, we considered the quarter of a mesh implant of size 3 cm and thickness 0.6 mm. It is modelled with shell elements (*S4R*: 4 node doubly curved, reduced integrated, hourglass control, and finite membrane strains). Tacks or sutures are used in practice to connect the mesh implant to the AW. In this model, an *embedding* constraint is used to couple the mesh with the hosting muscles. This constraint ensures that the translational degrees of freedom at the nodes of the embedded elements (lying within a host element) are eliminated. Their values are constrained by interpolating the corresponding translations of the host nodes.

As soft tissues, muscles can be modelled as hyperelastic materials such that the behaviour derives from a Helmholtz free-energy function. In this paper, we opted for an Arruda-Boyce potential, $\mathfrak {w}$, which includes a volumetric term, $\mathfrak {u}$, and an isochoric term, $\mathfrak {e}$, as follows:

1 $$\begin{aligned} \begin{array}{l} \displaystyle \mathfrak {w}(J, \varvec{C} ) = \mathfrak {u} (J) + \mathfrak {e} (J, \varvec{C} ) \end{array} \end{aligned}$$

where $\varvec{C} = \varvec{F}^T\varvec{F}$ is the right Cauchy-Green tensor, $\varvec{F}$ is the gradient of deformation, and *J* is the dimensionless dilatational ratio corresponding to the determinant of $\varvec{F}$. The three eigenvalues of $\varvec{F}$ denoted by $\{\lambda _X\}_{X=x,y,z}$ are the principal stretches that verify $\lambda _x \lambda _y \lambda _z= J = \text {det}\varvec{F} >0$. For isotropic materials, the principal stretches are sufficient to describe Helmholtz’s free-energy. Isotropy is adequate given that an individual layer of the abdominal wall musculature (e. g. internal oblique or rectus abdominis) tends to work as a single unit to achieve truncal movement in a specific direction. The two terms of Helmholtz’s free-energy (Eq. (1)) can be expressed as follows:

2 $$\begin{aligned} \begin{array}{l} \displaystyle \mathfrak {u} (J) = \frac{1}{D} \left( \frac{J^2 - 1}{2} - \text {ln} J\right) \text { and } \mathfrak {e} (J, \varvec{C} ) = \mu \sum _{i=1}^{5} \frac{C_i}{\lambda _m^{2i-2}} \left( \bar{I}_1^i -3^i\right) \end{array} \end{aligned}$$

where *D*, $\mu$, and $\lambda _m$ are parameters that can be calibrated experimentally, $\bar{I}_1$ is the first invariant ($\bar{I}_1 = \lambda _x^2 + \lambda _y^2 +\lambda _z^2)$ and the constants are $C_1=1/2$, $C_2=1/20$, $C_3=11/1050$, $C_4=19/7000$, and $C_5=519/673750$. The second Piola-Kirchhoff stress can be derived from Eq. (2):

3 $$\begin{aligned} \begin{array}{l} \displaystyle \varvec{S} = 2 \frac{\partial \mathfrak {u} (J) }{\partial \varvec{C} } + 2 \frac{\partial \mathfrak {e} (J, \varvec{C}) }{\partial \varvec{C} } = 2 \mathfrak {u}' (J) J_{,\varvec{C}} + 2 \frac{\partial \mathfrak {e} }{\partial \bar{I}_1 } \bar{I}_{1,\varvec{C}} \end{array} \end{aligned}$$

and Cauchy stress can be deduced from Eq. (3) as $\varvec{\sigma } = \varvec{F} \varvec{S} \varvec{F}^T/J$:

4 $$\begin{aligned} \begin{array}{l} \displaystyle \varvec{\sigma } = \mathfrak {u}' (J) \varvec{1} + 2\mu \sum _{i=1}^{5} \frac{i C_i}{\lambda _m^{2i-2}} \bar{I}_1^{i-1} \varvec{b} \end{array} \end{aligned}$$

where $\varvec{b} = \varvec{F} \varvec{F}^T$ is the left Cauchy-Green tensor. Hence, it can be verified that the hydrostatic pressure $p = \text {tr} \varvec{\sigma } /3 = \mathfrak {u}' (J) = \frac{1}{D} \left( J - J^{-1}\right)$, which means that the initial bulk modulus is $K = \frac{2}{D}$. For an incompressible material, the initial shear modulus can be obtained as $G =2 \frac{\partial \mathfrak {e} }{\partial \bar{I}_1 }$ at ${\bar{I}_1 = 3}$, which means that $\displaystyle G = 2 \mu \sum _{i=1}^{5} \frac{i C_i}{\lambda _m^{2i-2}} 3^{i-1}$. Consider a quasi-incompressible muscle subjected to a uniaxial that mimics the conditions of Cardoso (2012)^18^, the direction of loading $\varvec{e}_n$ is one of the principal directions such that $\lambda _n =\lambda$. From Eq. (4), it can be shown that under these conditions, $\sigma _{nn}$ is the only Cauchy stress that does not vanish and its expression is $\displaystyle \sigma _{nn}= 2\mu (\lambda ^2-\lambda ^{-1}) \sum _{i=1}^{5} \frac{i C_i}{\lambda _m^{2i-2}} \bar{I}_1^{i-1}$.

### Calibration and validation of the model

From the digitally reconstructed model of the abdominal muscles (Fig. 1), a subdomain around the umbilical area has been isolated, subjected to symmetry boundary conditions, and exposed to an IAP of 0.8kPa. This specific pressure has been selected because it represents an average IAP for the healthy weight group. We used, a non-linear hyperelastic constitutive model to describe the behaviour of the muscles based on the Helmholtz energy potential of Arruda-Boyce (see Eqs. 1 and 2). The material properties used for simulation and obtained by calibrating the hyperelastic model experimentally are summarised in Table 1.

**Table 1:** Materials properties used for simulation.

Specimens	Shear parameter, *G* (MPa)	Locking stretch, $\lambda _m$	Compressibility parameter, *D* (MPa$^{-1}$)
External oblique (EO)	0.01	1.01	92.3
Internal oblique (IO)	0.0065	0.85	142.011
Linea Alba (LA)	0.02	0.4	46.15
Rectus Abdominis (RA)	0.0052	0.775	177.5
transversus abdominis (TA)	0.0103	0.98	98.61

To calibrate the constitutive model, its theoretical response was compared to the experimental data of Cardoso^18^ who used cadaveric tissues and assessed the mechanical behaviour of various muscles (external oblique, internal oblique, rectus abdominis, and transversus abdominis). It is known that cadaveric tissues behave differently from living tissues as the *post mortem* biochemical and environmental changes can stiffen the muscular tissues. However, the difference in mechanical behaviour can be negligible especially if the samples were refrigerated, preserved in a B. Braun medical solution, and tested within 24 hours from collection to avoid degradation.

**Figure 3:**
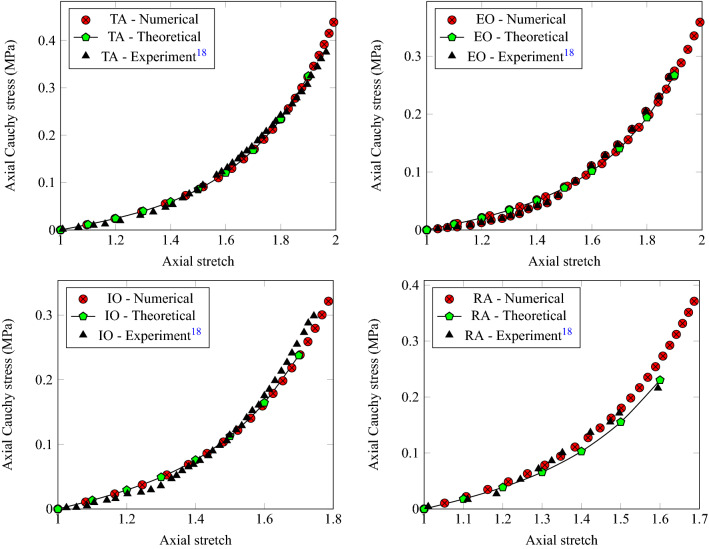
Calibration and validation of the model using the uniaxial experimental data of Cardoso conducted on specimens from abdomen muscles^18^.

To validate the constitutive behaviour discussed in the previous section, we developed a simple finite element model where we represented the tissues as incompressible cuboids of length $L_0 =$ 10 mm having a square cross section of size $a_0$ = 1 mm. The base of each cuboid was constrained axially and allowed to roll otherwise. The top was subjected to a surface traction defined per unit of unreformed area (first Piola-Kirchhoff stress or nominal stress). We compared the results of the model with experimental data and found that both the theoretical and numerical models accurately predicted the uniaxial behaviour of the tissues, as shown in Fig. 3. This validation provides evidence that our model can be used to accurately simulate the behaviour of soft tissues.

### Human and animal rights

No experiments were conducted on humans as part of this study.

## Results and discussion

**Figure 4:**
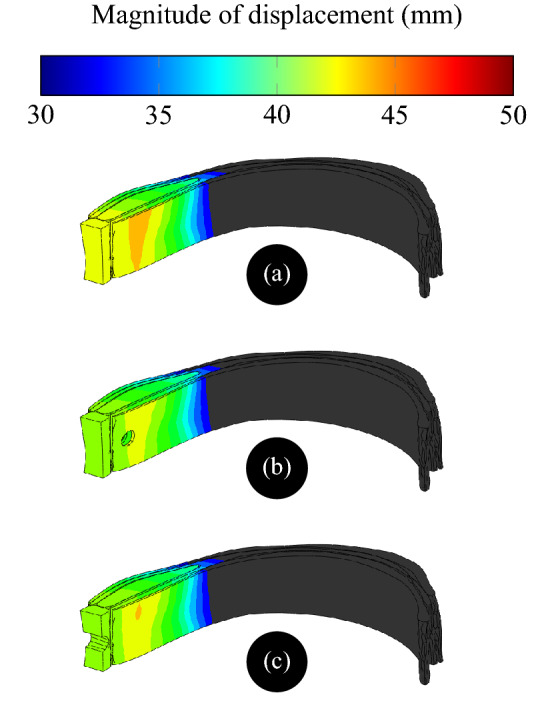
Magnitude of displacement in the AW subdomain (**a**) without hernia, (**b**) with incisional hernia, and (**c**) with umbilical hernia.

The FEM has been applied to calculate the response in displacement of the AW with and without hernia damage. The comparison in Fig. 4 indicates that the displacement decreases by 3% when hernia occurs in the AW. This decrease in displacement is explained by the absence of reaction to applied pressure where hernia has developed which leads to an overall lower cumulative force and thereby smaller displacement. This may also explain the protrusion of soft tissues through the hernia weakness as a reflection of pressure gradient.

**Figure 5:**
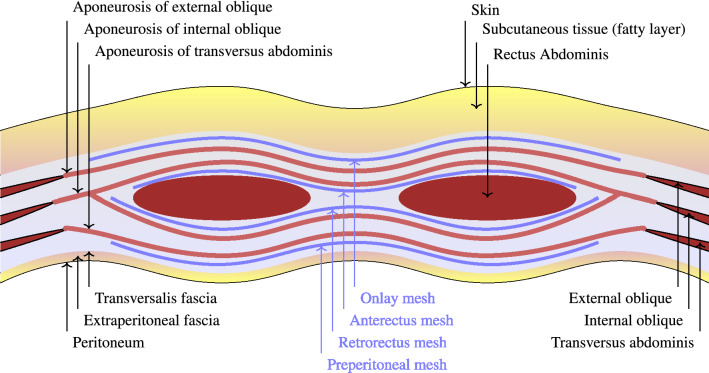
Mesh placements for ventral hernia repairs according to the international classification of abdominal wall planes^20^.

Hernia repair often involves the placement of a mesh, which confers tensile strength to the abdominal wall by promoting the formation of scar tissue. There has been a proliferation of mesh types with differing mesh materials, mesh weights (dependent on the weight and amount of material used) and porosity of the meshes. Despite the abundance of clinical studies investigating hernia repair with various types of mesh, there are a number of questions that have not been answered including the role of mesh in hernia repair and the optimal placement of the mesh within the layers of the abdominal wall^19^. Figure 5 describes the mesh insertion for ventral hernia repair according to the international classification of abdominal wall planes^20^.

### Incisional hernia

**Figure 6:**
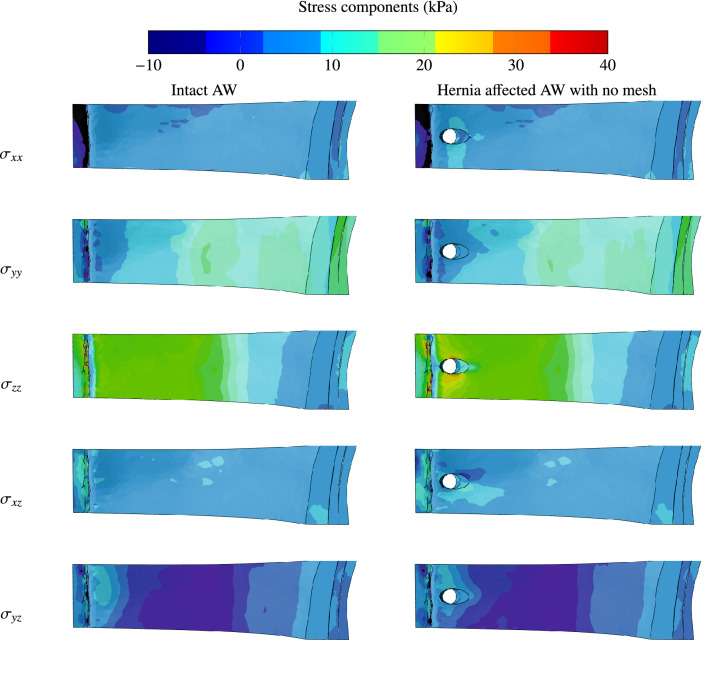
Contours of stress components for intact AW and hernia affected AW when no mesh is used to reinforce the damaged muscles. The sixth shear component stress tensor $\sigma _{xy}$ has been ignored because it is negligible.

In this study we focused on small post-surgical (i.e. incisional) or umbilical hernias that are both modelled as circular defects in the muscles of the AW. For incisional hernia, the damage zone is considered to traverse the consecutive muscles of the abdomen: EO, IO, RA and TA. Figure 6 depicts the distributions of stresses in the AW subdomain. These contours show that the horizontal stress $\sigma _{zz}$ is the most significant component near the plane of bilateral (or midline) symmetry. The stress component $\sigma _{yy}$ becomes more significant 90$^{\circ }$ around the vertical axis away from the midline. The shear stresses are all relatively smaller. This stress distribution implies that the hoop stress dictates the most critical state in the AW. In terms of stress distributions in the various muscles, the linea alba experiences the highest stresses in the studied domain. This is because the linea alba is the stiffest tissue in the AW. Another highly solicited muscle according to this numerical analysis is the transversus abdominis, which is directly exposed to internal pressure. Some of this pressure is transmitted to the internal oblique and the rectus abdominis through contact forces but after significant attenuation, as shown in Fig. 8.

**Figure 7:**
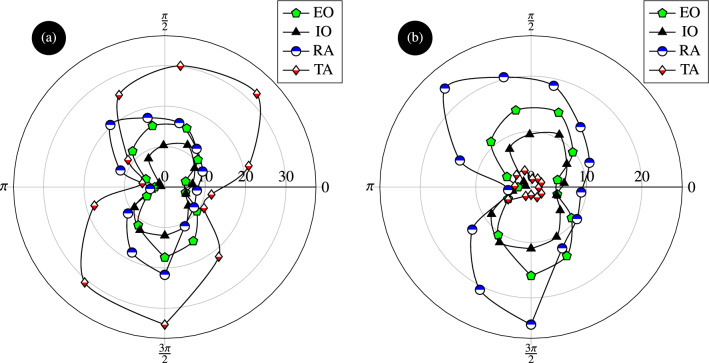
Distributions of equivalent stress (expressed in kPa) in the AW muscles affected by hernia (**a**) without reinforcement and (**b**) when a surgical mesh implant is used to reinforce the transversus abdominis. Hernia repair by mesh reinforcement reduces the magnitude of stresses and relieves their concentration.

The responses of an intact AW and a hernia affected AW with no reinforcing mesh were compared and the concentration of stresses around the hernia-damage zone and the amplification of stresses in this area were demonstrated, as shown in Fig. 6. The concentration and amplification of stresses prevail despite the smaller displacement that takes place when herniation occurs. Similarly, Fig. 7 depicts the magnitude of the equivalent stress around the hernia zone in rose diagrams (the origin of the polar coordinates corresponds to the centre of the hernia and the reference direction corresponds to the horizontal z-axis as shown in Fig. 4). The subfigures have two different scalings (Fig. 7a is from 0 to 30 kPa and Fig. 7a is from 0 to 20 kPa). The rose curves in Fig. 7a confirm that the highest stress is encountered in the transversus abdominis followed by the external oblique, while the stresses in the rectus abdominis and internal oblique are about five times smaller. This figure also indicates that the equivalent stress is anisotropic in the absence of reinforcement and that the TA experiences an equivalent stress of about 7.5 kPa in the horizontal polar direction and reaches 30kPa around the vertical polar direction. The stress concentration factor resulting from this stress anisotropy is 4 and it is attributed to the presence of hernia. The response in the presence of a surgical mesh implant used to reinforce the transversus abdominis is depicted by Fig. 7b. After hernia repair, no significant stress concentration can be perceived (the ratio of maximum over minimum stress is close to 1). In addition, stress amplification vanishes since the stress magnitude is about 5 kPa around the y-axis.

**Figure 8:**
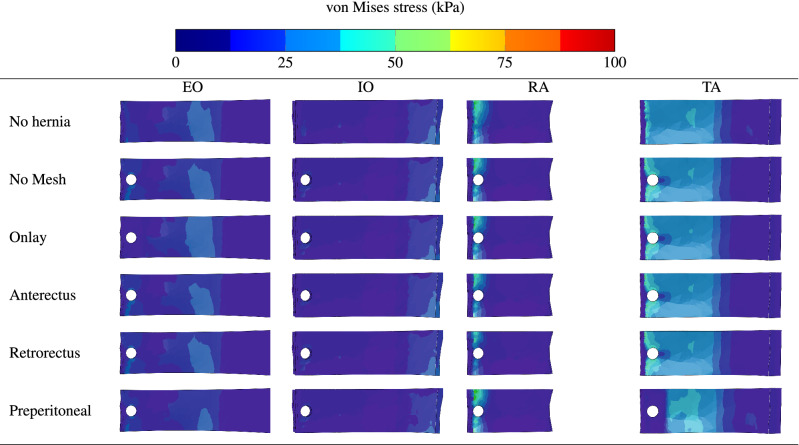
Contours of equivalent stresses in the different AW muscles in the absence of hernia (first row), in the absence of reinforcement and presence of hernia (second row), in the presence of hernia while surgical mesh implants are applied at the onlay (third row), anterectus (fourth row), retrorectus (fifth row), or preperitoneal (sixth row) planes.

Figure 8 shows the contours of equivalent stresses in the different AW muscles and in the absence of hernia (first row), in presence of hernia without repair (second row), and in the presence of hernia while surgical mesh implants are applied at the onlay plane (third row), anterectus plane (fourth row), retrorectus plane (fifth row), and preperitoneal plane (sixth row). The figure indicates that TA muscles are the most solicited in the AW irrespective of the presence or absence of reinforcement. This is because of their direct exposure to internal pressure. The stress distributions reveal that implanting a surgical mesh in the onlay, anterectus, or retrorectus planes does not improve the response of the AW to the applied internal pressure. However, the last row shows that reinforcing the TA muscle with a surgical mesh implant at the preperitoneal plane reduces the stresses significantly. Along with the results shown in Fig. 7, these contours indicate that for this hernia location, applying a preperitoneal mesh gives an optimum reinforcement and effectively reduces the stress concentration around the hernia damage zone.

**Figure 9:**
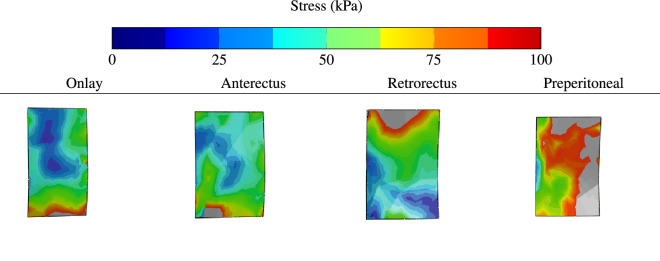
Equivalent stress distribution in the surgical mesh implants applied at the onlay, anterectus, retrorectus, or preperitoneal planes.

Examining the equivalent stress distributions in the surgical mesh implants applied at the onlay, anterectus, retrorectus, or preperitoneal planes (Fig. 9) shows that the maximum stresses occur in the TA mesh. This result is coherent with the conclusion drawn from Fig. 8. The high stress in the mesh suggests that it works effectively to protect its neighbouring muscles from stress concentration and amplification. Note that the maximum stresses obtained in this simulation are below the tensile strengths of commercially available composite and biological meshes reported by See et al.^21^.

### Paraumbilical hernia

Another significant case that is investigated in this study is paraumbilical hernia, which affects the linea alba (LA) near the region of the umbilicus. Deciding as to where mesh fabrics can be implanted is not well understood, thereby hernia repair can be in a sub-prime position if the mechanical response is unknown. Mesh placement in the retrorectus plane (Fig. 5) has become popular over the last years. This space lies between the rectus abdominis muscle and the posterior rectus sheath, which comprises the posterior leaf of the internal oblique aponeurosis and the transversus abdominis aponeurosis. In a large complex hernia repair, a posterior component separation procedure is sometimes needed^22^. In this manoeuvre, the posterior rectus sheath and transversus abdominis aponeurosis muscle are incised in order to reduce tension and the mesh is placed in the space between the rectus abdominis and internal and external oblique anteriorly and the posterior rectus sheath and peritoneal sac posteriorly.

**Figure 10:**
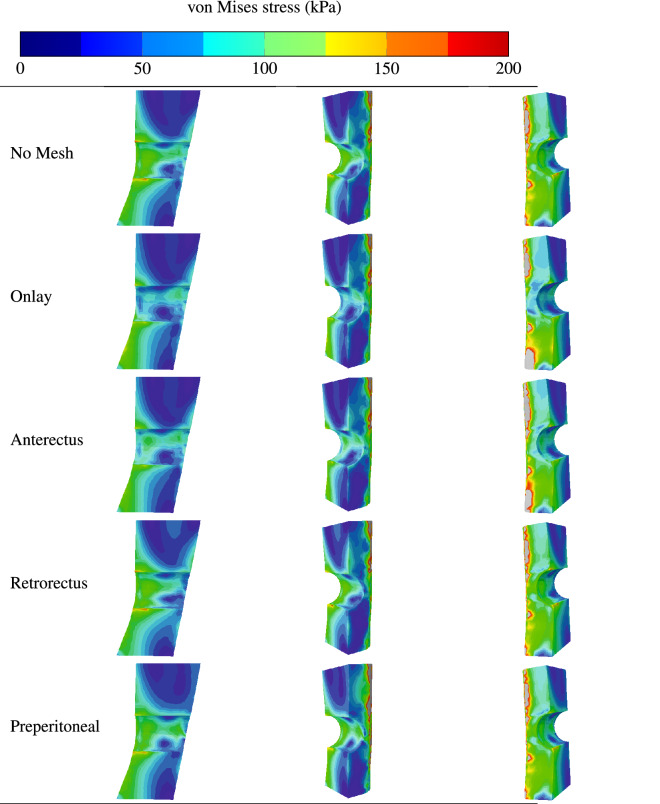
Contours of equivalent stresses in the linea albea affected by hernia when no mesh is applied (first row) and in the presence of a surgical mesh implants at the onlay plane (second row), anterectus plane (third row), retrorectus plane (fourth row), and at the preperitoneal plane (fifth row).

**Figure 11:**
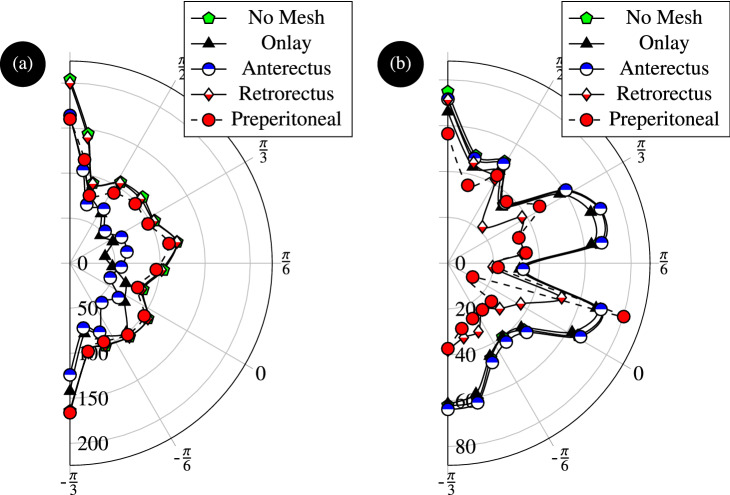
Distributions of equivalent stress (expressed in kPa) within the hernia damage zone around the (**a**) external and (**b**) internal interfaces of the linea albea with various mesh reinforcement positions .

The current numerical study sheds light on the optimum position through the abdominal tissues. Figure 10 depicts the contours of equivalent stress when no mesh is applied or when a mesh is applied at one of the inter-muscular interfaces. Although the stresses are high at the contact point between LA and the adjacent muscles, these singularities are not a source of concern as in practice contact areas are larger than the localised node-to-node interactions that concentrate stresses. The area of interest is at the periphery of the hernia and in its close vicinity. The contour plots indicate stress concentrations at the inner and outer surfaces of the LA muscles near the hernia; the magnitudes of the stresses are higher in the external areas. This result is confirmed by Fig. 11a,b indicating the stress distribution around the hernia damage zone at the external and internal faces.

Examining the effect of the mesh implant position on the stress distribution shows that the lowest stress distribution in the external ring is obtained for onlay mesh reinforcement (see Figs. 10 and 11a). Similarly, the lowest stress distribution in the internal ring is obtained when the reinforcement is placed in the preperitoneal plane (see Figs. 10 and 11b). The figures also show that the external stress decreases by about 50% when onlay mesh is implanted. Applying the mesh implant at the retrorectus plane reduces the stress by about 50% on average. Note that reinforcing the preperitoneal interface also reduces the stress in the internal zone but not as much as in the case of retrorectus interface reinforcement.

Other locations have practically no effect on the stress distribution, which means that they do not offer effective reinforcement. While the onlay and retrorectus planes are both suitable for mechanical reinforcement, it is more practical to place the mesh in the retrorectus plane to prevent the protrusion of soft tissues from the abdomen cavity. Clinical research proved that the retrorectus mesh placement is associated with reduced complication and recurrence rates^23^. For these reasons, placing the mesh implant at the retrorectus plane offers the best reinforcement in the case of umbilical hernia.

### Hernia growth

To assess the criticality of hernia size, we use the principles of fracture mechanics. Hence, the hernia is treated as a local crack of length *a* within the muscles. For a very short crack length, failure occurs when the ultimate stress $\sigma _u$ is reached. However, for long cracks, failure occurs at a stress $\sigma _f$ that is determined by measuring the fracture toughness. In the absence of a proper measurement of fracture toughness on human muscles, we consider the laboratory measurements of Taylor et al.^24^ conducted on porcine muscles and indicating that the apparent fracture toughness is $J_c = 2.5$ kJ/m$^2$. Note that for linear materials $K_c = \sqrt{J_c E}$ is known as the critical stress intensity factor. $J_c$ can be used for linear and non-linear materials while $K_c$ is meaningless when the behaviour is non-linear. Based on the definition of apparent fracture toughness, the critical hernia length is about

5 $$\begin{aligned} a_c = \frac{1}{\pi }\frac{J_c E}{\sigma _f^2} \end{aligned}$$

As shown in the stress contours discussed above, the AW investigated in this study experiences a nominal stress of no more than 0.1 MPa. For failure to occur at this stress in the EO, IO, RA, or TA, stress failure should be considered $\sigma _f = 0.1$ MPa. Table 2 shows the corresponding critical lengths if $J_c = 2.5$ kJ/m$^2$. Using Eq. (5), it can be seen that the most critical region is rectus abdominis, where cracks (or hernia damage zones) of critical size 4.1 cm or larger can propagate (i.e. grow). In the internal oblique, slightly larger cracks of sizes 5.2 cm can propagate. As for the external oblique and transversus abdominis, cracks of sizes 8 cm and 8.2 cm, respectively can propagate. The last column of Table 2 indicates the critical distance defined by

6 $$\begin{aligned} L = \frac{1}{\pi }\frac{J_c E}{\sigma _u^2} \end{aligned}$$

This distance can be interpreted as the size of a damage zone (e.g. hernia) that cannot influence the overall failure of a muscle (can be left in place without risk of crack propagation and therefore will not gain any significant benefit from a surgical repair of the hernia). Applying Eq. (6) shows that this critical size varies from 1.5 mm in the transversus abdominis to 7.8 mm in the rectus abdominis.

**Table 2:** Critical dimensions of cracks (or hernia defects) in abdominal muscles.

Specimen	*E* (MPa)		$\sigma _u$(MPa)		$a_c$ (cm)	*L* (mm)
	Mean	StDev	Mean	StDev		
External oblique	1.0	0.67	0.57	0.32	8	2.4
Internal oblique	0.65	0.29	0.39	0.19	5.2	3.4
Rectus abdominis	0.52	0.26	0.23	0.14	4.1	7.8
transversus abdominis	1.03	0.75	0.73	0.58	8.2	1.5

## Conclusion

A finite element model was developed to investigate the mechanical behaviour of a typical AW subjected to local damage due to hernia. The proposed modelling approach took into consideration the large deformation of abdominal muscles by incorporating non-linear geometrical changes and selecting an Arruda-Boyce hyperelastic constitutive model. The resulting stress distributions, hypothetical geometry of hernia and theory of fracture mechanics were used to assess the growth of hernias.

The proposed analyses revealed optimum locations of mesh implants enabling the effective reinforcement of hernia-affected abdominal muscles – locations that were previously selected empirically. The numerical results indicated that the optimum placement of mesh implants for incisional hernia is on the transversus abdominis muscles. As for paraumbilical hernia, retrorectus reinforcement of the linea alba proved to be more effective than preperitoneal, anterectus, and onlay implantations. Furthermore, this study provided an objective predictor of critical hernia size beyond which surgical intervention becomes necessary. In summary, the proposed workflow combined biomechanics and clinical knowledge to predict the response of a typical digitally reconstructed abdominal wall to internal pressure using non-linear geometrical changes, non-linear interactions and non-linear material behaviour.

Despite its potential, the proposed workflow has limitations that are essentially attributed to the lack of experimental data. The mechanical properties of muscles may differ from one patient to another. In addition, the loading constraints (e.g. intra-abdominal pressure) may also vary significantly with the health conditions of the patient (e.g. obesity, pregnancy). A model that takes into account such uncertainty might be more appropriate.

Future challenges in this area would require reliable testing of live and natural muscular tissues to predict the hyperelastic properties with more certainty and the fracture toughness under relevant loading conditions. As such, digitally constructed biomechanical models that are patient-specific have the potential to provide direct guidance to practicing surgeons, which can be instrumental to reduce hernia recurrence. These personalised constructed models will also account for patients’ compounding factors such body habitus and previous surgery.

## Data Availability

All data generated or analysed during this study are included in this published article. Any additional information relevant to the current study can be made available from the corresponding author on reasonable request.
